# Incidence of postsurgical pulmonary embolism and deep venous thrombosis: a single-center retrospective observational study

**DOI:** 10.1186/s40981-020-00328-5

**Published:** 2020-03-16

**Authors:** Hitomi Otsuka, Makoto Izumi, Eriko Ota, Noriaki Mochizuki

**Affiliations:** Department of Anesthesiology, Shinshu Ueda Medical Center, 1-27-21, Midorigaoka, Ueda City, Nagano, 386-8610 Japan

**Keywords:** Deep venous thrombosis and pulmonary embolism, Acute appendicitis, Complicated appendicitis, D-dimer, Enhanced computed tomography scan

## Abstract

**Background:**

Cancer is a risk factor for perioperative deep venous thrombosis and pulmonary embolism (DVT/PE). However, there is a paucity of data on non-malignant digestive diseases. In this study, we aimed to investigate the incidence of DVT/PE among patients, following surgery for acute appendicitis and other digestive diseases.

**Methods:**

We retrospectively reviewed the records of patients who underwent surgical procedures involving the digestive system between April 2018 and March 2019 attended by anesthesiologists (*n* = 536).

**Results:**

DVT/PE developed in seven patients (7/77, 9.1%, 95% confidence interval [CI] 3.7–17.8%) after surgery for acute appendicitis, and in six patients (6/83, 7.2%, 95%CI 2.7–15.1%) after elective surgery for colorectal cancer. Among the acute appendicitis group, six patients (6/30 20.0%) with complicated appendicitis (gangrenous or perforated appendicitis), and one patient (1/47 2.1%) with simple appendicitis showed postoperative DVT/PE. Patients with complicated appendicitis had a higher risk of DVT/PE than those with simple appendicitis with an odds ratio of 11.5 (95%CI 1.3–101.1).

**Conclusions:**

Although patients with acute appendicitis lack three of the risk factors for DVT/PE (cancer, long operative time, and older age), their 95% CI for the incidence of DVT/PE was comparable to that of patients undergoing elective surgery for colorectal cancer. Therefore, caution must be exercised during the perioperative period for preventing DVT/PE.

## Introduction

Deep venous thrombosis and pulmonary embolism (DVT/PE) could lead to life-threatening events during the perioperative period. It is well known that cancer patients are at a high risk of DVT/PE [1], because the tissue factor activates the coagulation pathway. There are several studies on the incidence of DVT/PE among cancer patients [2, 3]. However, there is a paucity of date regarding non-malignant digestive diseases as risk factors for DVT/PE.

We encountered seven cases of DVT/PE after appendectomy in our center last year. Li et al. observed endotoxin-induced activation of the extrinsic coagulation pathway in patients with acute appendicitis [4]. Other studies have identified inflammation as a risk factor for DVT [5]. Patients with acute appendicitis might be vulnerable to postoperative DVT/PE.

**Fig. 1:**
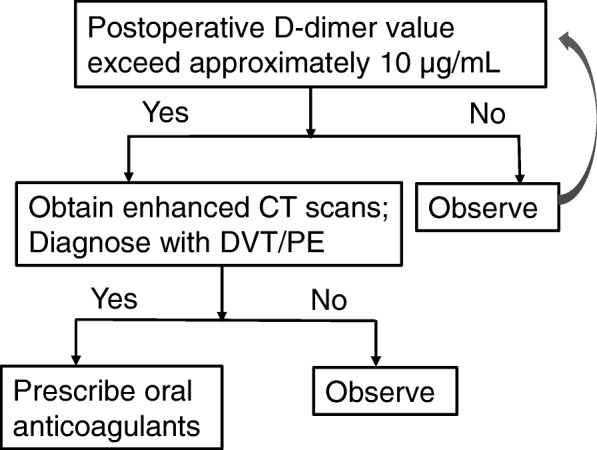
Diagnosis flowchart of DVT/PE in our center. DVT/PE, deep venous thrombosis and pulmonary embolism; CT, computed tomography

The incidence of DVT/PE differs depending on the diagnostic modality and criteria. In our center, the perioperative routine blood examination of patients who undergo surgeries for digestive diseases includes a D-dimer test. When the plasma D-dimer levels exceed approximately 10 μg/ml, we obtain enhanced computed tomography (CT) scans (Fig. 1).

The reported symptomatic PEs constitute just the tip of the iceberg. An analysis of the underlying asymptomatic DVT/PE cases will help reveal the risk factors. Hence, we conducted this study to compare the postoperative incidence of DVT/PE among patients with acute appendicitis and other digestive diseases.

## Methods

This study was approved by the ethical review board of the Shinshu Ueda Medical Center, and the need for informed consent from patients was waived. We retrospectively reviewed the medical records of patients who underwent digestive surgeries at Shinshu Ueda Medical Center from April 2018 to March 2019, attended by anesthesiologists (general anesthesia, neuraxial anesthesia, and local anesthesia). We excluded the following patients from the study: patients who already had PE or DVT on admission, and those who underwent a second operation during the hospital stay. We collected the following data: disease, operation method, age, sex, American Society of Anesthesiologist’s Physical Status (ASA-PS), body mass index, operative time, D-dimer value (preoperatively, postoperatively within 24 hours, and on the following days), and DVT/PE diagnosis. Continuous data are presented as mean (25th to 75th percentiles).

### Diagnosis of DVT/PE

DVT/PE was confirmed with enhanced CT scan when the plasma D-dimer value exceeded approximately 10 μg/ml. Enhanced CT scanning was performed with a 64-row multidetector scanner (Aquilion64; Canon Medical Systems Corporation, Ohtawara, Japan). A total non-ionic contrast material volume of 80 to 100 ml (iodine concentration, 300–370 mg/ml) was injected at the rate of 2.4 to 3.0 ml/s according to the patient’s weight. For pulmonary embolism, the scanning was started at 25 s after the start of injection, and the whole lung area was assessed. For deep venous thrombosis, the scanning was started at 210 s after the start of injection and the region from the diaphragm to the toes was investigated. In both areas, data were acquired with 0.5 mm thickness scans, and reconstructed for 2.0 mm-slice axial images, 5 mm-slice sagittal images, and 5 mm-slice coronal images. CT images were reviewed by a radiologist and the patient was accordingly diagnosed with DVT/PE. If a patient had any symptom of DVT/PE, we performed enhanced CT immediately, regardless of the D-dimer value. When the plasma D-dimer value did not exceed approximately 10 μg/ml and there were no symptoms, we performed physical examination and ultrasonography, if necessary, for observation.

### Studies

We reviewed the seven DVT/PE cases with acute appendicitis, including their symptoms, plasma D-dimer values, and CT findings.

We compared the incidence of DVT/PE between acute appendicitis and other disease categories. Then, we compared the background data (operative time and age) between patients with acute appendicitis and those with colorectal cancer, as asymptomatic DVT/PE incidence among colorectal cancer patients had been well studied [6, 7], and the number of operated cases are much higher in colorectal cancer than in other cancers. We thought DVT/PE incidence among colorectal cancer patients may provide a benchmark.

We further analyzed and compared the two categories of acute appendicitis, namely, complicated appendicitis and simple appendicitis. Categorization was made by intraoperative findings or postoperative pathological findings. We compared the incidence of DVT/PE, preoperative inflammatory biomarker (WBC, CRP, and Body temperature) levels, and plasma D-dimer values (preoperative and postoperatively within 24 h) between the two categories.

### Statistical analysis

The EZR (Saitama Medical Center, Jichi Medical University, Saitama, Japan) was used for statistical analyses. We compared the incidence of DVT/PE using the Fisher test with the Bonferroni post hoc test. We calculated the confidence interval of DVT/PE incidence with the Clopper-Pearson method. Continuous data were compared using the Mann-Whitney *U* test. *P* < 0.05 was considered statistically significant.

## Results

**Fig. 2:**
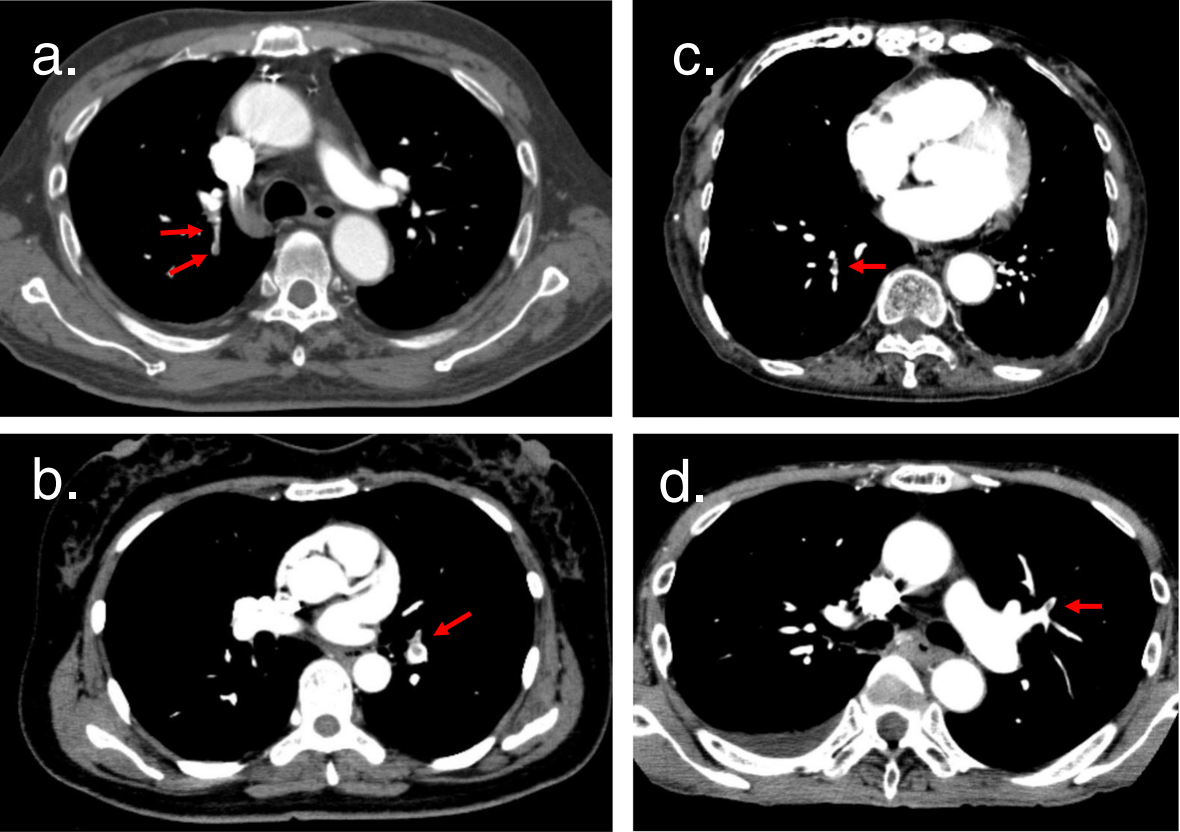
Enhanced CT images of pulmonary embolism in four patients with acute appendicitis. Red arrows indicate thrombi. **a** On POD3, plasma D-dimer level was elevated to 23.3 μg/ml. Enhanced CT revealed thrombus in right pulmonary artery. DVT was not found. **b** On POD6, the patient complained of right lower leg pain. Enhanced CT was performed without D-dimer test. Embolism in the left pulmonary artery and thrombi in the right popliteal vein and the right soleal vein were detected. **c** On POD6, plasma D-dimer level was elevated to 48.0 μg/ml. Some embolus was detected on both sides of the peripheral pulmonary arteries. DVT was detected in the left soleal vein. **d** On POD5, plasma D-dimer level was elevated to 33.2 μg/ml. Pulmonary embolism was detected in the upper right pulmonary artery. DVT was detected in the right popliteal vein. CT, computed tomography; POD, postoperative day; DVT, deep venous thrombosis

After appendectomy, seven patients were diagnosed with DVT/PE (Table 1). Only one patient showed lower leg pain. Others had no remarkable clinical findings. We showed some of the CT images in Fig. 2. After diagnosis, they were referred to a cardiologist and treated with oral anticoagulants at least for three months [8].

**Table 1:** Seven cases of deep venous thrombosis and pulmonary embolism (DVT/PE) after appendectomy

Case	Age (years)	Sex	Operative time (min)	Diagnosis day	Symptoms	Types of thrombosis	Pre D-dimer (μg/ml)	Post D-dimer (μg/ml)	Diag D-dimer (μg/ml)
Case 1	42	Female	83	2POD	None	PE	1.1	6.4	9.0
Case 2	65	Female	94	3POD	None	PE	0.7	6.1	10.5
Case 3	77	Male	80	3POD	None	PE	6.0	8.1	23.3
Case 4	86	Male	129	6POD	None	Proximal DVT	1.0	3.4	10.3
Case 5	47	Female	98	6POD	Lower leg pain	Distal DVT, PE	1.2	5.2	NA
Case 6	82	Female	59	6POD	None	Distal DVT, PE	3.5	6.3	48.0
Case 7	58	Male	74	5POD	None	Distal DVT, PE	18.2	25.1	33.2

*Pre* preoperative, *Post* postoperatively within 24 h, *Diag* diagnosis day, *POD* postoperative day, *PE* pulmonary embolism, *NA* D-dimer was not studied that day

Cases are arranged as per their order of occurrence

We calculated the incidence of DVT/PE in each disease categories (Table 2). DVT/PE incidence in acute appendicitis patients was 9.1% (95%CI 3.7–17.8). That incidence was significantly higher than that in the elective hernia patients. DVT/PE incidence in colorectal cancer patients was 7.2% (95%CI 2.7–15.1). There were no statistically significant differences between the colorectal cancer patients and other disease categories.

**Table 2:** Incidence of perioperative deep venous thrombosis and pulmonary embolism (DVT/PE) after digestive surgery

Disease	DVT or PE cases/total number (*n* = 520)	% (95%CI)
Hepatic cancer, pancreatic cancer	5/22	22.7 (7.8–45.4)^b^
Colorectal perforation	2/9	22.2 (2.8–60.0)
Acute appendicitis	7/77	9.1 (3.7–17.8)^a^
Gastric cancer	2/22	9.1 (1.1–29.2)
Colorectal cancer (elective surgery)	6/83	7.2 (2.7–15.1)
Small bowel obstruction	2/28	7.1 (0.9–23.5)
Acute cholecystitis	2/41	4.9 (0.6–16.5)
Strangulated hernia (femoral or inguinal)	0/16	0 (0–20.6)
Gastrointestinal perforation	0/7	0 (0–41.0)
Hernia (elective surgery; femoral or inguinal)	0/133	0 (0–2.7)^a^^,^^b^
Gallstone	0/45	0 (0–7.9)
Others	2/37	5.4 (0.7–18.2)

*DVT* deep venous thrombosis, *PE* pulmonary embolism, *CI* confidence interval

Sixteen patients were excluded from the study (patients who already had PE or DVT on admission, or those who underwent a second operation during the hospital stay)

Perforated colorectal cancer cases were included in “colorectal perforation”

^a^Acute appendicitis vs hernia (elective surgery), *p* = 0.049

^b^Hepatic cancer, pancreatic cancer vs hernia (elective surgery), *p* = 0.003

No statistically significant differences were seen among other categories (Fisher test, Bonferroni post hoc test)

95% confidence intervals were calculated with the Clopper-Pearson method

Operative time and a patient’s age are known risk factors for DVT/PE after a surgical operation. The operative time was significantly shorter in acute appendicitis patients than in colorectal cancer patients (68 [55–89] vs 184 [156–236] min; *P* < 0.001). The acute appendicitis patients were significantly younger than colorectal cancer patients (40 [14–58] vs 76 [68–83] years; *P* < 0.001).

Among the acute appendicitis group, six patients (6/30 20.0%) with complicated appendicitis and one patient (1/47 2.1%) with simple appendicitis had postoperative DVT/PE. Patients with complicated appendicitis had a higher risk of DVT/PE than those with simple appendicitis with an odds ratio of 11.5 (95%CI 1.3–101.1). Among preoperative inflammatory biomarkers, CRP value was significantly higher in complicated appendicitis patients than in simple appendicitis patients (Table 3).

**Table 3:** Comparison of the inflammatory biomarkers and plasma D-dimer levels between complicated appendicitis and simple appendicitis

Variable	Complicated (*n* = 30)	Simple (*n* = 47)	*P* value
Temperature (°C)	37.2 (36.7–38.3)	37.1 (36.7–37.7)	0.32
WBC (10^2^/μl)	12,700 (10,300–15,650)	12,600 (9500–14,500)	0.59
CRP (mg/dl)	9.4 (4.0–18.4)	1.0 (0.4–2.4)	< 0.001*
Pre D-dimer (μg/ml)	1.15 (0.58–2.65)	0.40 (0.13–0.70)	< 0.001*
Post D-dimer (μg/ml)	3.45 (1.75–6.10)	0.50 (0.30–1.30)	< 0.001*

*WBC* white blood cell count, *CRP* C reactive protein, *Pre D-dimer* plasma D-dimer level examined preoperatively, *Post D-dimer* plasma D-dimer level examined postoperatively within 24 h

Continuous data are presented as median (25th to 75th percentile)

*P* values were calculated using the Mann-Whitney *U* test

*P < 0.05

## Discussion

In this retrospective study, DVT/PE occurred in seven and six patients after surgery for acute appendicitis and colorectal cancer. Furthermore, patients with complicated appendicitis showed a higher risk of DVT/PE than those with simple appendicitis.

The incidence of asymptomatic DVT/PE after colorectal surgery is reported in some literatures. Routine screening with Doppler ultrasonography revealed that the DVT incidence after colorectal surgery was 3.0% [6]. Another study reported that DVT/PE incidence among laparoscopic colorectal surgery patients without anticoagulant prophylaxis was 5.1% using enhanced CT screening for all patients [7]. Higher incidence of DVT/PE is reported in laparoscopic surgery for gastrointestinal cancer as 18.3% [9], and 24.3% in major abdominal surgery [10]. In this study, the incidence of DVT/PE after colorectal surgery was 7.2%.

To our knowledge, DVT/PE incidence after surgery for acute appendicitis has not been reported yet. This neglect could be attributed to the absence of the three risk factors, cancer, long operative time, and older age, in the case of acute appendicitis. However, in our study, the 95% confidence interval for DVT/PE incidence in acute appendicitis patients was comparable to that in colorectal cancer patients (Table 2).

In further analysis, complicated appendicitis showed significantly higher incidence of DVT/PE than simple appendicitis. Inflammation was previously considered to be a risk factor of DVT/PE [5]. Patients with complicated appendicitis were reported to have higher plasma IL-6 level than those with simple appendicitis [11]. Elevated IL-6 might have induced DVT [12] in those patients. Further study is needed to confirm this theory.

Among DVT/PE cases after surgery for acute appendicitis, the D-dimer values gradually increased with the passing day (Table 1). We do not know whether this reflected the resolution of the fibrinolysis suppression or a growing clot in the veins. If it is because of thrombosis progression a few days after the operation, enhancing the postoperative rehabilitation may be helpful for the prevention of DVT/PE.

The limitations of this study were that this was a single-center study, technical errors might have occurred while drawing blood (slow drawing causes coagulation cascade in the needle), the timing of blood sampling was not strictly determined, and our attending physicians used a high D-dimer cutoff value of approximately 10 μg/ml for ordering enhanced CT based on their clinical experiences, instead of conventionally proposed 0.5 μg/ml [13]. Therefore, some patients with DVT/PE might not have been diagnosed.

Future studies should evaluate other non-malignant inflammatory diseases as risk factors for DVT/PE and explore their associated thromboses and hemostasis mechanisms. In addition, the adequate perioperative D-dimer cutoff value for digestive surgeries should be studied.

## Conclusions

In summary, our data demonstrated that complicated appendicitis could be a risk factor for postsurgical DVT/PE. Among DVT/PE cases after surgery for acute appendicitis, the D-dimer values increased with the passing day. Thus, caution must be exercised during the perioperative period for preventing DVT/PE.

## Data Availability

The datasets generated and/or analyzed during the current study are available from the corresponding author on reasonable request.

## Abbreviations

DVT/PE: Deep venous thrombosis and pulmonary embolism
CT: Computed tomography
ASA-PS: American Society of Anesthesiologist’s Physical Status
POD: Postoperative days
